# Exposure Assessment of Infants to Aflatoxin M_1_ through Consumption of Breast Milk and Infant Powdered Milk in Brazil

**DOI:** 10.3390/toxins8090246

**Published:** 2016-08-31

**Authors:** Angélica T. Ishikawa, Cássia R. Takabayashi-Yamashita, Elisabete Y. S. Ono, Artur K. Bagatin, Fabiana F. Rigobello, Osamu Kawamura, Elisa Y. Hirooka, Eiko N. Itano

**Affiliations:** 1 Department of Pathological Sciences, State University of Londrina, P.O. Box 10.011, 86057-970 Londrina, Paraná, Brazil; fabianarigobello@gmail.com (F.F.R.); itano@uel.br (E.N.I.); 2 Department of Food Science and Technology, State University of Londrina, P.O. Box 10.011, 86057-970 Londrina, Paraná, Brazil; kcia_rt@yahoo.com.br (C.R.T.-Y.); hirooka@uel.br (E.Y.H.); 3 Department of Biochemistry and Biotechnology, State University of Londrina, P.O. Box 10.011, 86057-970 Londrina, Paraná, Brazil; eysono@hotmail.com; 4 Food Hygiene Laboratory, Faculty of Agriculture, Kagawa University, 761-0795 Miki-cho, Kagawa, Japan; arturbagatin@yahoo.com (A.K.B.); kawamura@ag.kagawa-u.ac.jp (O.K.)

**Keywords:** exposure, mycotoxin, milk, infant, safety, carry-over

## Abstract

Aflatoxin M_1_ (AFM_1_) is an important biomarker that can be used to evaluate aflatoxin exposure in both humans and animals. The aim of this study was to evaluate the exposure degree of infants to AFM_1_ through consumption of breast milk and infant powdered milk in Brazil. For this purpose, the estimated daily intake (EDI) for infants was calculated based on the AFM_1_ levels analyzed in 94 breast milk (BM) samples collected in Southern Brazil, and 16 infant powdered milk (IPM) samples commonly commercialized in Brazil. AFM_1_ was detected in 5.3% (*n* = 5) and 43.8% (*n* = 7) of BM and IPM samples, with mean levels of 0.003 ng/g and 0.011 ng/g, respectively. All the IPM samples showed AFM_1_ levels lower than those established by the Brazilian guidelines (5 ng/g), and in most of the samples (81.25%) levels were below the maximum limit tolerated by the European Commission (0.025 ng/g). The EDI of AFM_1_ for infants aged zero to 12 months old showed values from 0.018 to 0.069 ng/kg body weight/day for BM, and 0.078 to 0.306 ng/kg body weight/day for IPM. Hazard index (HI) values for BM and IPM were less than one, except for IPM intended for infants up to one month. In conclusion, the exposure of infants to AFM_1_ was low, but continuous monitoring of mycotoxin levels is essential to minimize infant health risk.

## 1. Introduction

Human breast milk (BM) is recommended for the first six months of life for infants along with continued breastfeeding for up to two years. Breastfeeding promotes the mother-child relationship and ensures better growth and development of the newborn, providing nutrients, antibodies and leukocytes [1]. However, many infants and children do not receive optimal food, wherein only 38% of infants aged zero to six months worldwide and 41% of Brazilian infants of the same age are exclusively breastfed [1,2,3].

There is a special concern for infants about some trace toxins, such as aflatoxin M_1_ (AFM_1_) which is a monohydroxylated derivative of aflatoxin B_1_ (AFB_1_), present in milk. Aflatoxins (AFs) are fungal secondary metabolites that are primarily produced by *Aspergillus flavus* and *A. parasiticus*. Although AFM_1_ carcinogenicity is about 10-fold less than that of AFB_1_, AFM_1_ carcinogenicity has been shown in experimental animals. AFM_1_ is classified as Group 2B, possibly carcinogenic to humans [4]. It has been suggested that children are more susceptible than adults to acute hepatotoxicity resulting from ingestion of aflatoxin and effects of toxicants due to their higher metabolic rate, lower body weight, immature metabolic pathways, and incomplete development of tissues and organs [4,5,6]. Children exposed to AFM_1_ may be underweight and stunted during infancy and thus become more prone to infectious diseases [4,7].

An AFM_1_ biomarker can be used to evaluate aflatoxin exposure through diet for both humans and animals. Human exposure to AFs occurs through the intake of contaminated agricultural products or the consumption of products from animals that were fed with contaminated feed. This contamination may occur by fungal growth during harvest or improper storage [8]. Because the Brazilian milk market is characterized by big companies purchasing raw milk from different suppliers [9], companies should use high quality feedstock. Food contaminated with AFB_1_ is metabolized in animals and the human liver into AFM_1_ by Cytochrome P450–associated enzymes, and then distributed in serum and excreted into milk and urine. In mammals, AFM_1_ can be detected in milk within 12 h after the ingestion of AFB_1_ [10,11]. The carry-over of AFB_1_ in feed metabolized into AFM_1_ in milk for dairy cows was usually 1%–2% for low-yielding cows (<30 kg milk yield/day) and up to 6% for high-yielding cows (>30 kg milk yield/day) [12].

Several authors have reported the presence of the AFM_1_ biomarker in breast milk around the world such as in Nigeria [13], Turkey [14], Egypt [15], Iran [16], and Italy [17], with a wide variation in AFM_1_ contamination (0.13 to 1.730 ng/L). In Brazil, low AFM_1_ levels in breast milk have been reported [18,19]. Toxicological evaluation of aflatoxin over food intake is essential to any risk evaluation and important for determining the relationship observed in humans and exposure to aflatoxin [20,21]. There are few data available on AFM_1_ intake by infants (zero to 12 months old) in the Brazilian literature [21,22], and around the world most of the Estimated Daily Intake (EDI) data were for children (one to eight years old) [23,24,25].

Many countries have set or proposed guidelines for the maximum tolerated AFM_1_ levels in milk and milk products because of their risk for human health. The Brazilian Health Surveillance Agency [26] has not established maximum AFM_1_ levels for infant products, although the maximum limits for fluid milk and powdered milk should be 0.5 and 5 ng/g, respectively. A technical regulation on the Maximum Tolerated Limit for Mycotoxins in Food has not been defined regarding whether the fluid milk is raw or pasteurized. The European guidelines established the maximum AFM_1_ levels in both raw milk (0.05 ng/g) and infant formulae (0.025 ng/g) [27]. Based on the potential hazard to infant (zero to 12 months old) health due to carry-over of the aflatoxin biomarker (AFM_1_) into milk, the aim of this study was to evaluate the exposure of infants to AFM_1_ through consumption of breast milk and infant powdered milk. The data of this study are relevant for the Brazilian population, considering that breast milk donors in Southern Brazil have similar food consumption as other regions of Brazil, and powdered milk samples for infants were from brands commonly commercialized in Brazil.

## 2. Results

### 2.1. Description of Food Consumption by Lactating Mothers

Among the foods ingested by the 92 lactating mothers during 24 h before the BM was collected, 94.5% of the mothers consumed rice, 81.5% consumed beans, and 76.1% consumed bread. Only a small percentage of the food ingested by participants was derived from peanut products (3.3%), nuts (2.2%), dried prunes, and ginger (1.1%).

### 2.2. Method of Evaluation of AFM_1_ Analysis in Milk

The Limit of detection (LOD) was 0.003 ng/g and 0.004 ng/g, and the Limit of quantification (LOQ) was 0.016 ng/g and 0.021 for infant powdered milk (IPM) and breast milk (BM), respectively. The mean recovery rates of spiked BM (*n* = 3) at levels of 0.025, 0.05 and 0.5 ng of AFM_1_/g were 110.25% ± 5.57% (Relative Standard Deviation (RSD) = 5.05), 97.21% ± 1.96% (RSD = 2.02) and 88.35% ± 1.01% (RSD = 0.01), respectively. The recovery test values for IPM at the same levels were 98.21% ± 6.43% (RSD = 6.54), 77.92% ± 1.34% (RSD = 1.72) and 71.67% ± 1.15% (RSD = 1.60). The equation was *y* = 1090832.98*x* − 10692.71, with a coefficient of determination (R^2^) greater than 0.99 (Table 1).

### 2.3. Breast Milk and Infant Powdered Milk Analysis

Table 2 shows the AFM_1_ levels in 94 BM samples. AFM_1_ was detected in 5.3% (*n* = 5) of the BM samples, with levels ranging from 0.013 to 0.025 ng AFM_1_/g (mean of 0.018 ± 0.005 ng/g). Seven of the 16 IPM samples (43.8%) were contaminated with AFM_1_ (LOD: 0.03 ng/g), and the levels ranged from not detectable (n.d.) to 0.046 ng/g, with a mean of 0.024 ± 0.001 ng/g (Table 3). Nine samples of IPM produced in Brazil showed contamination levels ranging from n.d. to 0.046 ng/g (0.024 ± 0.01 ng/g). Furthermore, seven samples of imported IPM showed contamination levels from n.d to 0.034 ng/g (0.024 ± 0.01 ng/g). There was no significant difference in the mean AFM_1_ levels for BM and IPM (*p* > 0.05).

### 2.4. Estimated Daily Intake (EDI) and Hazard Index (HI) for AFM_1_ for Infants through Breast Milk and Infant Powdered Milk Consumption

The mean AFM_1_ levels in BM and IPM calculated according to International Programme on Chemical Safety/Global Environment Monitoring System (IPCS/GEMS) criteria [28] were 0.003 and 0.011 ng/g, respectively (Table 4). Considering the mean AFM_1_ levels, EDI values for BM ranged from 0.018 to 0.067 ng/kg b.w./day for males and 0.019 to 0.069 ng/kg b.w./day for females (Table 4). For IPM, the EDI values ranged from 0.078 to 0.296 for males and 0.084 to 0.306 ng/kg b.w./day for females. Concerning HI values for BM, the values ranged from 0.09 to 0.35 for both sexes, and for IPM they ranged from 0.39 to 1.53.

## 3. Discussion

Human exposure to AFB_1_ through diet can be estimated from the aflatoxin concentration in foods and the amounts that are consumed. Alternatively, the determination of the aflatoxin biomarker in body fluids, such as AFM_1_ in milk and urine, is more reliable for this type of evaluation [31]. In this study, the BM samples showed low AFM_1_ levels (n.d. (not detectable) to 0.025 ng/g). Similarly, in a study conducted by Iha et al. [32], only two of 100 BM samples showed AFM_1_ contamination (0.3 and 0.8 ng of AFM_1_/mL). Andrade et al. [19] analyzed 224 samples from the BM bank in the Distrito Federal, Brazil, and detected no contamination with AFM_1_ (LOQ = 0.01 ng/mL).

In this study, food consumption by lactating mothers was in agreement with the traditional Brazilian diet. According to Brazilian Institute of Geography and Statistics (IBGE) [33], the items with the highest average daily consumption for the Brazilian population are beans (177.1 g) and rice (131 g). The prevalence of bread consumption ranges from 53.4% to 73.6% in all the regions of Brazil (northern, northeastern, southeastern, southern and center-western), while for oleaginous foods such as nuts and peanuts, it ranges from 0.5% to 1.4%.

It is well known that humid and warm conditions, genetics of fungi and plants, and food storage conditions are some factors that influence mycotoxin production. Aflatoxins have been detected in a variety of agricultural commodities, but the most pronounced contamination is related to corn, peanuts, cottonseed, and tree nuts [4]. In Brazil, aflatoxin occurrence and levels are low in beans and rice [34,35]. However, aflatoxin occurrence has been reported in corn and peanuts [31,36,37]. AFB_1_ frequency and contamination levels are low in corn and derivatives from Paraná State [38,39]. In this study, it was not possible to make a statistical correlation between the presence of AFM_1_ in breast milk and peanut consumption due to the low number of contaminated samples. However, the AFM_1_ detected at low levels in BM samples was probably due to the AFB_1_ and AFM_1_ contamination levels in the food consumed of local origin by mothers before the BM was collected.

Polychronaky et al. [40] reported that the consumption of grains, milk and dairy products, meat, fish, vegetables, cotton seed oil, dried fruits and peanuts by lactating mothers did not show any significant associations with AFM_1_ in BM. Andrade et al. [19] also reported that it was not possible to correlate nut and peanut consumption by BM donors and AFM_1_ in milk, because AFM_1_ was not detected. The authors emphasized that aflatoxin intake (0.06–0.08 ng/kg b.w./day) and cancer risk (0.0006–0.0009 cancers/year/10^5^ individuals) values were low for the population of the Federal District, center-western Brazil, showing no risk for the population. In Italy, four out of 86 samples of BM were contaminated with AFM_1_, and no relation to food consumption was found. However, the lactating mother whose milk showed high AFM_1_ levels (0.14 ng/mL) had consumed a large amount of corn meal–based foods in substitution of cereal-based food such as rice, pasta, bakery products and breakfast cereals [17]. In other regions of the world, such as Africa, a correlation between the ingested food and AFB_1_ metabolites in body fluids has been observed [13,15]. This correlation could be greater in cases of acute aflatoxicosis arising from intakes of high AFB_1_ levels by Asian and African populations, whose common diet is based on cereals and grains [41].

BM has many benefits for the infant as it protects against gastrointestinal infections, reduces newborn mortality, promotes healthy growth and development; it also contains a proper balance of fats, carbohydrates, vitamins and proteins. Despite the advantages of BM, in some situations mothers cannot breastfeed; thus, powdered milk is an alternative for providing the required nutrients to newborns and infants [2,3]. Among the 16 IPM samples analyzed in this study, seven samples were contaminated with AFM_1_ (0.012 to 0.046 ng of AFM_1_/g), and three of them exceeded the maximum level allowed, which is 0.025 ng of AFM_1_/g [27]. However, in Brazil, there is no legislation for AFM_1_ in IPM. Moreover, the current Brazilian regulation is less stringent than the European Legislation for powdered milk, and therefore, the three aforementioned samples were within the allowed limits, i.e., 5 ng of AFM_1_/g [26]. Two samples of IPM contaminated with AFM_1_ were produced in Argentina, while five samples of raw milk from Brazilian producers (Southeastern and Southern regions) were produced in the Northwest of São Paulo State (Table 3). AFM_1_ levels in raw milk samples from different dairy farms in Brazil ranged from 0.012 to 0.725 µg/kg [9,42].

Because AFM_1_ is thermostable (120 °C) and is not readily destroyed or removed by chemical and physical treatments, monitoring AFB_1_ levels in animal feeds is essential to minimize AFM_1_ contamination in IPM [43]. Thus, a maximum level of 50 ng of AFs (AFB_1_ + AFB_2_ + AFG_1_ + AFG_2_)/g in the raw materials has been allowed for animal feed in Brazil [44]; and the maximum content allowed of AFB_1_ for complete feeds has been established at 0.005 ng of AFB_1_/g for dairy animals such as cattle, sheep and goats by European Commission [45].

Risk assessment (hazard identification, hazard characterization, exposure assessment and risk characterization) is the scientific evaluation of estimating human exposure to xenobiotic compounds through food consumption and it provides a link between possible hazards in the food chain and the risks reflected in human health [46,47]. Although there are differences in the frequency of consumption and in variations in the volume of milk consumed by infants, it is possible to estimate the exposure of infants to AFM_1_ using the EDI. It is noteworthy that male and female infants from zero to 12 months of age show differences in body weight and milk consumption; thus, the EDI level is affected by these factors. The EDI values of AFM_1_ for infants decrease as the age is increases, showing the susceptibility of younger infants (Table 4).

In this study, EDI values (0.018–0.306 ng AFM_1_/kg b.w./day) were lower than those reported by other authors in Brazil. Oliveira et al. [22] reported an EDI of 3.7 ng AFM_1_/kg b.w./day for a four-month-old infant weighing 6 kg, representing a daily intake of 22 ng of AFM_1_. In other studies, the EDI ranged from 0.106 to 1.04 ng AFM_1_/kg b.w./day for children (23 kg) who consumed 400 mL of powdered milk with 0.006 to 0.061 ng AFM_1_/g, respectively [9,21]. There are few data available on the AFM_1_ intake by infants (zero to 12 months old) in the Brazilian literature, and around the world most EDI data were for children (one to eight years old). EDI values from different countries (Spain, Argentina and Thailand) ranged from 0.16 to 3.70 ng AFM_1_/kg b.w./day [23,24,25]. Recently, Ismail et al. [48] reported EDI values for male and female children from Pakistan ranging from 1.68 to 5.45 AFM_1_/kg b.w./day, and the highest EDI value (5.45 ng AFM_1_/kg b.w./day) was 17.8-fold higher than that obtained in this study (Table 4). According to The Joint Food and Agriculture Organization/World Health Organization (FAO/WHO) Expert Committee on Food Additives (JECFA), the average dietary intake of AFM_1_ for adults is 3.5 ng/person/day based on the Latin American diet [43].

Based on studies in male Fischer rats, Kuiper-Goodman [49] has determined a No Observed Effect Level (NOEL) for AFM_1_ of <2.5 µg/kg body weight/day. Furthermore, the author also proposed the dose of 0.2 ng/kg body weight/day as a “safe dose”, i.e., 50% of the animals would have developed tumors (TD_50_) dividing by a large safety factor of 50,000. In this study, the Hazard Index (HI) values for BM and IPM were less than one, except for IPM intended for infants up to one month old for which the HI value was more than one, indicating a risk to infant health.

Early childhood exposure to aflatoxins is difficult to detect by clinical signals, but it may be critically determinant for immediate and later health effects. Therefore, continuous monitoring of aflatoxin occurrence in foods, particularly in IPM, is necessary because this food is an important alternative for infants’ food nutrition in situations where mothers cannot breastfeed. Nevertheless, breastfeeding is important and must be encouraged for children up to two years of age when possible.

## 4. Conclusions

In this study, the exposure of infants to AFM_1_ through BM was low (HI < 1) indicating that lactating mothers had a low exposure to AFB_1_. However, the HI value for newborns to AFM_1_ through IPM was greater than one. Therefore, continuous monitoring of AFM_1_ and establishing a maximum AFM_1_ limit in the Brazilian legislation for infant formulas are required due to the higher susceptibility of infants to mycotoxin contamination compared to adults.

## 5. Material and Methods

### 5.1. Experimental Section

#### 5.1.1. Breast Milk and Infant Powdered Milk Samples

#### 5.1.1.1. Breast Milk Samples

Healthy lactating mothers were invited to participate in this study in three hospitals in Londrina City, Brazil (State University of Londrina Teaching Hospital, Evangelical Hospital and Municipal Maternity), on alternate days from June to August 2013. The State University of Londrina Teaching Hospital attends patients from 250 cities in Paraná State and more than 100 cities in other States, mainly São Paulo, Mato Grosso, Mato Grosso do Sul and Rondônia. The exclusion criteria for the lactating mothers were malnutrition, fever, and diseases of the breast. A total of 2.7% of the Brazilian population (20 years or more) is underweight [50], and 25% women who breastfeed are affected by mastitis [51].

A total of 94 lactating mothers agreed to participate in this study and the BM was collected at least 10 days after parturition. After collection, the BM was transported to laboratory in a coldbox, and it was immediately frozen at −14 °C, lyophilized and kept at −14 °C until analysis. The project was approved by Human Ethics Committee of State University of Londrina (CEP/UEL, 159/2012), and all the volunteers were informed about the study protocol, a written informed-consent agreement was signed and a food inquiry/questionnaire was applied.

The lactating mothers were instructed to complete a 24 h recollection reporting all foods ingested on the day (24 h) before BM collection in order to identify potential AFB_1_ sources [10,11,52]. The foods commonly contaminated with AFB_1_ and AFM_1_, and consumed by lactating mothers were grouped as cereals and derivatives (rice, corn, bread, biscuit, cake, spaghetti), milk and derivatives (milk, cheese, yogurt), and others (bean, paçoca-product derived from peanuts, chocolate, nuts, dried prunes, ginger). The results of food consumption from each group were expressed by percentage in relation to 92 lactating mothers because two questionnaires were incompletely filled.

#### 5.1.1.2. Infant Powdered Milk Samples

A total of 16 samples of Brazilian milk-based infant formulae from different industries, belonging to three brands commonly commercialized in Brazil, were purchased in markets of Londrina City from March to April 2013. Then they were vacuum packed and stored until analysis. The samples were recommended for infants aged zero to 12 months, nine samples were produced in Southeastern Brazil and seven samples were imported from the United States of America, Germany and Argentina.

### 5.2. Immunoaffinity Column (IAC) for Clean up of Milk Samples

The immunoaffinity column based on monoclonal antibody was manufactured to clean up the milk samples. For this purpose, Hybridoma AM.3, secreting monoclonal antibody (mAb) for AFM_1_ derived from SP2/0-Ag14 myeloma cell line and BALB/c spleen [53], was cultivated in Hybridoma Serum Free Medium (HSFM, Gibco, Life Technologies, Grand Island, NY, USA) at 37 °C, 5% CO_2_ (Sanyo, Osaka, Japan). The IC_50_ value of Hybridoma AM.3 to AFM_1_ was 6.1 pg/mL, and its cross-reactivities (%) were 0.2 (AFM_2_), 0.002 (AFG_1_), <0.0006 (AFG_2_), 0.003 (AFB_1_), <0.0006 (AFB_2_). For Immunoaffinity Column (IAC) manufacture, an amount of 3 mL of Affi-gel 10 (Bio-Rad Laboratories, Hercules, CA,USA) was washed with ultra-pure water at 4 °C (three-fold volume of gel) and added to 4 mg of anti-AFM_1_ mAb/mL of gel, and gently mixed for 16 h at 25 °C. A solution of 1 mol/L monoethanolamine-HCl pH 8.0 was added to block the gel and mixed for 1 h at 25 °C. The gel was washed with 0.01 mol/L Phosphate Buffered Saline (PBS) pH 7.4, and an amount of 0.3 mL of coupled gel was placed in a polypropylene column (Muromac^®^ Mini-column, Omuta, Fukuoka, Japan) for AFM_1_ extraction, and further analysis.

### 5.3. AFM_1_ Extraction from Breast Milk and Infant Powdered Milk

For BM, an aliquot of 10 mL of 0.01 mol/L PBS pH 7.4 was added to 2 g of lyophilized BM and shaken at 200 rotation per minute (rpm) for 15 min. Subsequently, the volume was adjusted up to 16 mL with 0.01 mol/L PBS pH 7.4. For IPM, a volume of 20 mL of 0.01 mol/L PBS pH 7.4 was added to 4 g of IPM and 1.8 g of NaCl and shaken at 200 rpm for 15 min, and the volume was adjusted up to 25 mL with 0.01 mol/L PBS pH 7.4. After the BM and IPM samples were centrifuged at 1670× *g* for 20 min, the fat layer was removed, and the supernatant was filtered using two glass fiber filters (GA-200 followed by GA-55, Advantec, Tokyo, Japan). The AFM_1_ extraction by IAC was carried out with 10 mL of 0.01 mol/L PBS pH 7.4, followed by 10 mL of reconstituted BM or IPM. The IAC was washed with 5 mL of PBS 0.01 mol/L PBS pH 7.4 and 5 mL of distilled water. The water inside the IAC was removed by adding air (30 mL). The elution was performed with 10 mL of CH_3_OH:CH_3_CN (1:9), and the eluate was dried in a rotary evaporator (Eyela, Tokyo, Japan). In this study, the rapid drying time was an important factor to maintain stability of AFM_1_ for analysis.

### 5.4. Method Evaluation

The AFM_1_ detection method was validated considering the following parameters: Limit of Detection (LOD, signal:noise ratio, >3:1), Limit of Quantification (LOQ, signal:noise ratio, >10:1), recovery test, repeatability and linearity. Milk samples with non-detectable AFM_1_ levels were used for spiking. The recovery rates of spiked BM and IPM were measured at levels of 0.025, 0.05 and 0.5 ng AFM_1_/g on three different days. The repeatability measured by relative standard deviation was checked from replicates of reconstituted BM and IPM samples in the recovery study. Linearity was determined from three calibration curves with four levels from 0.025 to 5 ng AFM_1_/mL.

### 5.5. Aflatoxin M_1_ Analysis by High Performance Liquid Chromatography (HPLC)

The dried extract was resuspended in 1 mL of mixture of ultra-pure H_2_O: CH_3_OH: CH_3_CN (60:30:10, *v*/*v*/*v*). An aliquot of 10 µL was injected into HPLC equipped with an oven (CTO-10A), pump (LC20AD), degasser (DGU-20A3), auto sampler (SIL-20AHT), fluorescence detector (RF-20AXS), and reversed-phase column (XR-ODS/C8/Phenyl, 3 mm × 100 mm, 2.2 µm, Shimadzu, Kyoto, Japan). The excitation and emission wavelengths were 365 nm and 435 nm, respectively. The mobile phase was H_2_O: CH_3_OH: CH_3_CN (60:30:10, *v*/*v*/*v*) at a flow rate of 0.5 mL/min at 50 °C, with a total run time of 10 min [54]. The AFM_1_ concentration was expressed in ng of AFM_1_/g.

### 5.6. Estimated Daily Intake (EDI) and Hazard Index (HI) for AFM_1_ for Infants

The EDI of AFM_1_ for infants was estimated from relationships between the daily milk intake, i.e., the volume of milk consumed by infants, the mean AFM_1_ contamination in BM and IPM samples, and average body weight for males and females. The milk consumption considered was 590, 642, 560, 452 mL for ages one week, one month, six months and 12 months, respectively [29]. For the volume of milk consumed by infants, it was considered that 1 g lyophilized BM corresponded to 8 mL reconstituted BM, and 4.52 g IPM was required to prepare 30 mL milk according to the manufacturers. The male and female average weight were 3.3 and 3.2 kg for infants aged one week, 4.5 and 4.2 kg for one month, 7.9 and 7.3 kg for six months, and 9.6 and 8.9 kg for 12 months [30]. The EDI of AFM_1_ was calculated according to the following formula and expressed in ng/kg of body weight/day (ng/kg b.w./day) [55].

$$\text{EDI}\left(\text{ng}/\text{kg b}.w./\text{day}\right)=\frac{\text{daily milk intake}\times {\text{mean AFM}}_{1}\text{ concentration}}{\text{average body weight}}$$

In this study, AFM_1_ contamination in BM and IPM samples was evaluated, and values below the LOD were assumed to be 0.5 × LOD or 0 according to the IPCS/GEMS [28] criteria adopted to estimate mycotoxin contamination when values lower than the LOD are observed. These criteria consider a proportion of results lower than the LOD, first when all the results are over the LOD, the true means are calculated; second, when the proportion of results below the LOD is lower than 60%, the mean is calculated by replacing those observations with 0.5 × LOD; third, when the proportion of results lower than the LOD is equal to or over 60%, the mean is calculated replacing first those observations by 0 and second replacing them with the LOD. In this study, the proportion of results lower than the LOD was over 60% for BM, and the proportion of results lower than the LOD was below 60% for IPM. The Hazard index (HI) for AFM_1_ was determined by dividing the EDI by TD_50_ (the dose at which 50% of the animals would have developed tumors) divided by a safety factor of 50,000, and the proposed value is 0.2 ng/kg b.w. [47,49]. HI higher than 1.0 indicates risk to consumers [56].

### 5.7. Statistical Analysis

Data was analyzed by the Statistica software (version 7.0, 2004, StatSoft, Tulsa, OK, USA) and the AFM_1_ levels were expressed in ng/g. Before the analysis, homogeneity of variances was carried out using the Levene’ test and data distribution was analyzed by the Shapiro-Wilk test, and the positive BM and IPM samples were compared by the Test T.

## Figures and Tables

**Table 1:** Recovery of AFM_1_ in breast milk and infant powdered milk.

AFM_1_ Added (ng/g)	Breast Milk		Infant Powdered Milk	
	Recovery (%)	RSD (%)	Recovery (%)	RSD (%)
0.025	110.25 ± 5.57	5.05	98.21 ± 6.43	6.54
0.05	97.21 ± 1.96	2.02	77.92 ± 1.34	1.72
0.5	88.35 ± 1.01	0.01	71.67 ± 1.15	1.60

BM, LOD: 0.004 ng/g; LOQ: 0.021 ng/g; IPM, LOD: 0.003 ng/g; LOQ: 0.016 ng/g; BM: Breast Milk; IPM: Powdered Milk; LOD: Limit of detection; LOQ: Limit of quantification; SD: Standard Deviation; RSD: Relative Standard Deviation.

**Table 2:** Distribution of aflatoxin M_1_ levels in naturally contaminated breast milk samples from lactating mothers of Paraná State, Brazil.

Breast Milk	Aflatoxin M_1_		
*n*	Positive Samples (%)	Range (ng/g)	Mean ^a^ (ng/g)
89	-	n.d.	-
3	100	0.013–0.015	0.014 ± 0.001
2	100	0.022–0.025	0.024 ± 0.002
Total: 94	5.3	n.d.–0.025	0.018 ± 0.005

The distribution of aflatoxin M_1_ levels was divided in: (a) <LOD; (b) between LOD and LOQ; and (c) >LOQ; LOD: 0.004 ng/g, LOQ: 0.021 ng/g; ^a^ Mean of positive samples; LOD: Limit of detection; LOQ: Limit of quantification; *n*: number of samples; n.d.: not detectable.

**Table 3:** Aflatoxin M_1_ levels in infant powdered milk retailed in Brazil.

Infant Powdered Milk		Aflatoxin M_1_		
Product Origin	*n*	Positive Samples (%)	Range (ng/g)	Mean ^a^ (ng/g)
National	9	55.6	n.d.–0.046	0.024 ± 0.01
Imported	7	28.6	n.d.–0.034	
Total:	16	43.8	n.d.–0.046	0.024 ± 0.01

LOD: 0.003 ng/g; LOQ: 0.016 ng/g; ^a^ Mean of positive samples; LOD: Limit of detection; LOQ: Limit of quantification; *n*: number of samples; n.d.: not detectable.

**Table 4:** Age group, milk consumption, average weight and AFM_1_ estimated daily intake by infants through naturally contaminated breast milk and powdered milk *.

Age	Milk Consumption (mL) [16]	Average Weight (kg) [17]		Estimated Daily Intake (ng/kg b.w./Day)			
				BM		IPM	
		M	F	M	F	M	F
1 week	590	3.3	3.2	0.067	0.069	0.296	0.306
1 month	642	4.5	4.2	0.054	0.057	0.236	0.253
6 months	560	7.9	7.3	0.027	0.029	0.117	0.127
12 months	452	9.6	8.9	0.018	0.019	0.078	0.084

* Considering International Programme on Chemical Safety/Global Environment Monitoring System (IPCS/GEMS) criteria [28], the mean of AFM_1_ contamination in BM and IPM were 0.003 and 0.011 ng/g, respectively; [29] Brazil, 2005; [30] SBP, 2009; M: male; F: female; b.w.; body weight.
